# Longitudinal coupling between depressive symptoms and divided attention during transcranial magnetic stimulation in treatment-resistant depression

**DOI:** 10.1017/S1092852926100935

**Published:** 2026-05-13

**Authors:** Mao-Hsuan Huang, Yee-Lam E. Chan, Jun-Ding Zhu, Wei-Chung Mao, Wei Yuan, Yia-Ping Liu, Li-Chi Chen, Feng-Chang Yen, Wei-Shen Huang, Tung-Ping Su

**Affiliations:** 1 Department of Psychiatry, General Cheng Hsin Hospital, Taipei, Taiwan; 2 School of Medicine, College of Medicine, National Yang Ming Chiao Tung University, Taipei, Taiwan; 3 Department of Occupational Therapy, College of Medical Science and Technology, Chung Shan Medical University, Taichung, Taiwan; 4 Occupational Therapy Room, Chung Shan Medical University Hospital, Taichung, Taiwan; 5 Institute of Brain Science, National Yang Ming Chao Tung University, Taipei, Taiwan

**Keywords:** Attention, cognitive function, depression, naturalistic, transcranial magnetic stimulation

## Abstract

**Background:**

Cognitive dysfunction is a core feature of major depressive disorder. During transcranial magnetic stimulation (TMS) for treatment-resistant depression (TRD), the temporal relationship between symptom changes and cognitive performance remains unclear. We examined depressive symptoms and cognitive trajectories during TMS and tested whether time-varying cognitive change was associated with symptom trajectories.

**Methods:**

This retrospective naturalistic study included 89 patients with TRD who completed 20 sessions of left dorsolateral prefrontal cortex TMS (high-frequency repetitive TMS or intermittent theta-burst stimulation). Depression severity (Hamilton Depression Rating Scale [HAMD-17]) and computerized attentional control measures from the Test of Attentional Performance were assessed at baseline, day 10, and day 20. Generalized estimating equations examined time effects and time-adjusted symptom-cognition associations. Exploratory analyses evaluated whether early cognitive change was associated with subsequent symptom improvement. Multiple comparisons were controlled.

**Results:**

Depressive symptoms decreased significantly (p < 0.001), with response and remission rates of 65.2% and 51.7%. Several attentional indices showed significant time effects; however, after false discovery rate correction, only visual divided-attention commission errors were independently associated with depressive severity beyond time effects. Person-mean centering indicated that this association was driven by within-person change (B = 0.209, p < 0.001), not between-person differences. Greater early reduction in visual divided-attention commission errors was associated with larger overall symptom improvement (B = 0.172, p = 0.016).

**Conclusion:**

Within-person changes in divided attention tracked depressive symptom trajectories during TMS. Attentional control measures may therefore help track symptom-cognition dynamics during TMS in real-world clinical settings.

## Highlights

1. Assessment at three time points instead of only pre–post TMS comparison.
2. Depressive symptoms declined during a 20-session TMS course in TRD.
3. Several attentional indices showed significant time effects.
4. Visual divided-attention errors were linked to symptom severity beyond time.
5. Coupling was driven by within-person change, not between-person change.
6. Early divided-attention improvement correlated with greater symptom reduction.

## Introduction

Major depressive disorder (MDD) is a leading cause of disability worldwide and is increasingly recognized as a heterogeneous condition extending beyond affective symptoms alone.^1^ In addition to depressed mood and loss of interest, cognitive impairments, encompassing deficits in attention, executive function, and working memory, are common in acute episodes and may persist despite symptomatic remission, contributing to functional disability, reduced treatment adherence, and elevated relapse risk.^2^ Accordingly, cognitive dysfunction has emerged as a key treatment target in depression because it is a major determinant of functional impairment and quality of life.^3^ ^–^^5^

Existing pharmacological treatments for depression have demonstrated limited and inconsistent effects on cognitive outcomes. Antidepressant medications primarily target mood symptoms, and cognitive improvement often lags or remains incomplete, particularly in patients with treatment-resistant depression (TRD).^4^ ^,^^6^ There has been growing interest in non-pharmacological neuromodulation approaches, such as transcranial magnetic stimulation (TMS), as potential interventions to address both affective and cognitive dimensions of depression.^7^ TMS targeting the dorsolateral prefrontal cortex (DLPFC) is an established treatment for TRD and is mechanistically relevant to cognitive control networks.^8^ ^,^^9^

However, whether a therapeutic TMS course produces reliable cognitive enhancement in depression remains uncertain.^10^ ^–^^12^ Recent meta-analyses of randomized, sham-controlled trials have found no robust, generalized cognitive benefit of active TMS over sham across broad domains in unipolar depression, including attention, working memory, psychomotor speed, language, and executive function.^10^ Subsequent task-level analyses have suggested that TMS-related cognitive effects may be modest and domain-specific rather than generalized, with small improvements observed on measures emphasizing psychomotor speed, visual scanning, and set-shifting.^12^ Notably, these cognitive changes appear independent of mood improvement, implying partially distinct trajectories from antidepressant response.^13^ These mixed findings highlight unresolved questions regarding which cognitive processes change during a real-world therapeutic course and whether such changes track symptom improvement rather than simply co-occurring with time. Most studies rely on pre–post comparisons, limiting insight into the temporal relationship between cognitive and clinical change during TMS treatment.^12^ In addition to baseline cognitive function, within-treatment cognitive change may provide informative signals for predicting antidepressant response.^14^ ^,^^15^

To address these gaps, we conducted a retrospective, naturalistic study of patients with TRD who completed a standard 20-session course of either high-frequency repetitive TMS (HF-rTMS) or intermittent theta-burst stimulation (iTBS) targeting the left DLPFC, with repeated assessments at baseline, midcourse, and end of treatment. Rather than testing cognitive efficacy, the present study was designed to characterize the within-treatment trajectories of depressive symptoms and cognitive performance and to examine whether time-varying cognitive changes are associated with symptom change after accounting for repeated assessment over time. Given the mixed task-level signals in prior literature and the feasibility constraints of repeated testing in routine care, we focused on DLPFC-relevant attentional control indices (working memory updating, divided attention, and response inhibition) that can be assessed repeatedly with minimal burden and reduced practice effects.^16^ ^–^^18^ This study aimed to (1) describe longitudinal changes in depressive severity and cognitive performance during a therapeutic TMS course and (2) test whether within-treatment changes in cognitive performance are coupled with symptom trajectories independent of time effects. We hypothesized that DLPFC-mediated attentional control indices would show the strongest time-adjusted association with depressive symptom change.

## Methods

### Study design, participants, and procedure

This retrospective observational study included patients with TRD who received consecutive treatment with either HF-rTMS or iTBS at Cheng Hsin General Hospital (Taipei, Taiwan) between January 2019 and September 2023. All treatments were delivered in a naturalistic, open-label clinical setting. The study was approved by the Institutional Review Board of Cheng Hsin General Hospital (22-2958-104), and the requirement for written informed consent was waived due to the retrospective design. Because this was a retrospective naturalistic study, treatment allocation was not randomized and no sham control was included.

**Figure 1.**
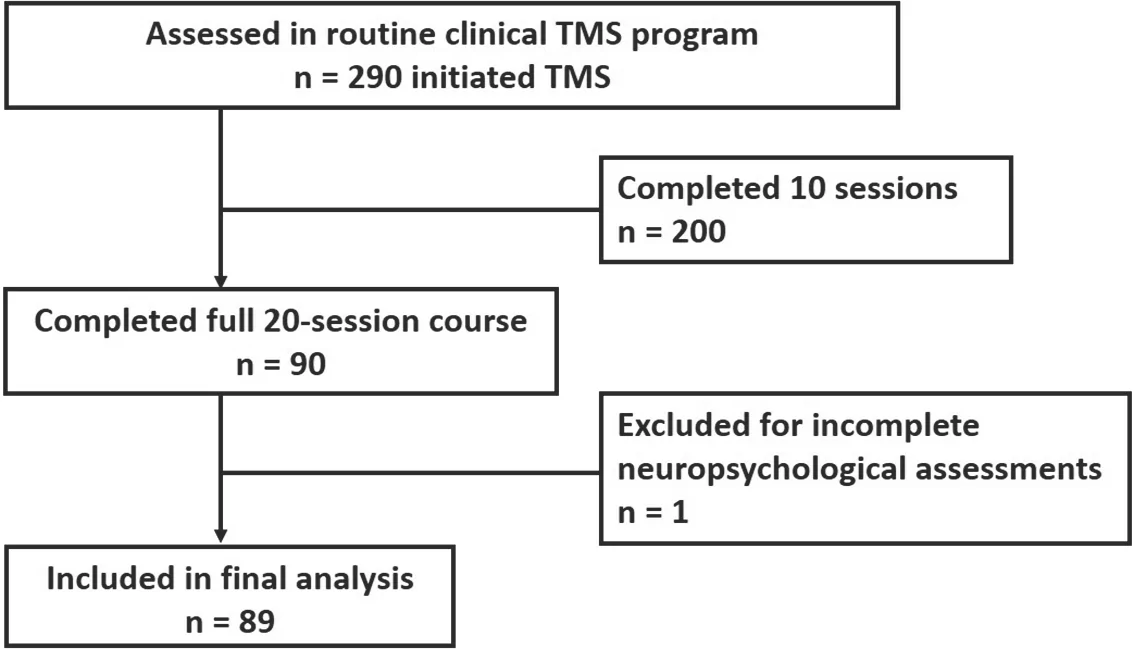
A STROBE flow diagram of participant selection.

Clinician-rated interviews and computerized neuropsychological testing were part of routine clinical care in the TMS program. During the study period, 290 patients initiated TMS treatment at our center. Of these, 200 (69%) completed 10 sessions, and 90 (31.0%) completed a 20-session course (Figure 1). The analytic cohort was defined a priori as patients who were clinically scheduled to receive a full 20-session course and who completed all scheduled neuropsychological assessments. Patients who completed only 10 sessions were not considered for inclusion a priori, because this would provide only 2 assessment time points (baseline and day 10) and a shorter treatment exposure that was not expected to yield the intended therapeutic effects on mood and cognition within this study protocol. No patient discontinued treatment prematurely due to adverse events, symptom worsening, or lack of efficacy. One patient did not complete the day 10 and day 20 neuropsychological assessments and was therefore excluded from the final analyses. Because the present analyses were restricted to patients who completed the full 20-session treatment course and all scheduled assessments, the findings reflect a treatment-completer cohort rather than the entire population initiating TMS.

To further evaluate potential selection bias related to treatment completion, sensitivity analyses were conducted comparing patients who completed 10 sessions with those who continued to complete 20 sessions (Supplementary Table S1). First, baseline demographic and clinical characteristics were compared between groups. Second, early clinical outcomes on day 10 were compared, including response and remission rates, to assess whether continuation to 20 sessions was primarily driven by early treatment response. Response and remission rates did not differ between groups (response: *χ^2^ p* = 0.701; remission: *χ^2^ p* = 0.355), suggesting that continuation to 20 sessions was not primarily determined by early clinical improvement and reducing the likelihood of confounding by indication and survivorship bias. Third, to examine whether treatment continuation was associated with systematic differences in early cognitive trajectories, baseline cognitive performance and day 10 cognitive outcomes were compared between groups across all test of attentional performance (TAP) indices. These analyses were intended to assess whether the completer cohort showed evidence of differential baseline cognitive function or early cognitive change that could bias longitudinal analyses. Importantly, real-world implementation studies have shown that adherence to TMS is strongly influenced by treatment burden, including out-of-pocket cost, daily time commitment, and logistical barriers, rather than clinical response alone.^19^

All inpatients and outpatients referred to the TMS unit were evaluated by an experienced psychiatrist (Prof. Su) to determine treatment indications and contraindications. MDD was diagnosed using the Mini-International Neuropsychiatric Interview and DSM-5 criteria. TRD was defined as failure to respond to at least 2 antidepressant trials administered at adequate doses and durations (≥8 weeks), verified through detailed interview or chart review.^20^

Patients aged 18–80 years were eligible. Exclusion criteria included bipolar disorder, psychotic disorders, intracranial or intracochlear metallic implants, pregnancy, unstable epilepsy, and substance use disorder within 6 months prior to screening. Concomitant psychotropic medications were permitted but were kept stable throughout the TMS treatment course. Specifically, during the treatment course, no initiation, discontinuation, or dose adjustment of primary antidepressants, antipsychotics, or mood stabilizers was allowed; only pro re nata hypnotics were permitted when clinically indicated. The choice between HF-rTMS and iTBS was determined through shared decision-making between psychiatrists and patients. Treatment sessions were delivered on weekdays; missed sessions were rescheduled whenever possible and were not delayed by more than 2 days. Adverse events were routinely monitored and recorded.

### Brain stimulation procedures

TMS was administered using a Magstim Rapid^2^ stimulator (Magstim, Sheffield, UK). Resting motor threshold was determined as the minimum stimulation intensity required to elicit visible contraction of the contralateral abductor pollicis brevis muscle in 5 of 10 consecutive trials. The left DLPFC was targeted in all patients by positioning a figure-eight coil in the parasagittal plane 5.5 cm anterior to the motor hotspot. For HF-rTMS, stimulation was delivered at 10 Hz at 100% resting motor threshold, with 4-second trains and 26-second inter-train intervals. Each session included 40 trains (1600 pulses) and lasted approximately 20 minutes. For iTBS, stimulation consisted of 1800 pulses delivered as triplet bursts at 50 Hz repeated at 5 Hz (2 seconds on, 8 seconds off) at 80% resting motor threshold. All patients received treatment 5 days per week for a total of 20 sessions.

### Clinical and neuropsychological assessments

#### Clinical assessment

Demographic variables including age, sex, and education level were collected. Depression severity was evaluated using the Hamilton Depression Rating Scale (HAMD-17) at baseline, after 10 treatment sessions (day 10), and after completion of 20 sessions (day 20). Treatment response was defined as a reduction of ≥50% in HAMD-17 total score from baseline, and remission was defined as a HAMD-17 total score < 7 at the end of the 20-session treatment course. When treatment sessions were delayed, follow-up assessments were conducted after completion of all 20 sessions. In addition to the HAMD-17 total score, changes in the HAMD-17 item 10 (suicidal ideation) and clinical global impression (CGI)-severity were examined as exploratory descriptive clinical indicators because of their clinical relevance in routine TMS care.

#### Neuropsychological assessment

Cognitive performance was assessed using the TAP,^21^ including n-back (working memory), Go/No-Go (inhibitory control), and divided-attention tasks. Assessments were performed at baseline prior to TMS initiation, on day 10 (week 2), and after completion of 20 sessions (week 4). To reduce practice effects, standardized practice trials were administered prior to each testing session. Primary cognitive outcomes included the number of correct responses, omission errors, and commission errors across TAP subtests. For a detailed explanation of the cognitive test procedure, please refer to the Supplementary Method.

### Statistical analysis

Generalized estimating equations (GEE) were used to examine longitudinal changes in depression severity and neuropsychological performance across the 3 assessment time points. GEE were selected to account for within-subject correlations in repeated measurements and to provide population-averaged estimates. An autoregressive correlation structure of order 1 (AR-1) was specified as the working correlation matrix. Age, sex, treatment group (HF-rTMS vs iTBS), and psychotropic medication use were included as covariates in all models. Separate GEE models were first conducted to evaluate the overall time effect on HAMD-17 total scores and cognitive performance indices. Further GEE models were constructed to evaluate longitudinal associations between depressive symptom severity and each cognitive performance index during the TMS course. Two hierarchical models were specified: In model 1, the HAMD-17 total score was modeled as a function of time while adjusting for age, sex, treatment group, and psychotropic medication use. In model 2, the TAP indices were added as a time-varying covariate to model 1 to examine the independent association between cognitive performance and depressive symptoms beyond the effect of time. The Benjamini–Hochberg false discovery rate (FDR) procedure was used to control for multiple comparisons arising from the evaluation of multiple TAP indices. To disentangle between-person and within-person effects of divided-attention performance, visual divided-attention commission error (DAV_error) was decomposed using a person-mean centering approach. For each participant, a subject-specific mean of DAV_error across the 3 time points was calculated (DAV_error_mean) to represent the between-person effect. A within-person deviation score (DAV_error_within) was then computed as the difference between the observed value at each time point and the subject-specific mean. Both components were simultaneously entered into the GEE model to examine whether depressive symptom severity was associated with stable between-person differences or within-person fluctuations in divided-attention performance. To facilitate the interpretation of the time-adjusted association between cognitive performance and depressive symptoms, model-based predicted HAMD-17 scores were derived from the GEE model. Predicted values were calculated across the observed range of DAV_error while holding time and other covariates constant. These predicted HAMD-17 values were plotted to visually depict the association between DAV_error and depressive symptom severity. In an exploratory analysis, we examined whether early cognitive change was associated with subsequent clinical improvement. Percentage change in depressive symptoms was calculated from baseline to day 20 as: (baseline HAMD total score−day20 HAMD total score)/baseline HAMD total score. Early change in TAP performance was calculated from baseline to day 10 as: (baseline TAP−day10 TAP)/baseline TAP. Linear regression analysis was used to examine the association between early cognitive improvement and subsequent symptom improvement, adjusting for age, sex, treatment, and baseline HAMD total score. The level of significant difference was set at a *p*-value <0.05. Statistical analysis was performed using SPSS 29.0 (SPSS Inc., Chicago, IL, USA).

## Results

A total of 89 patients with TRD who completed a full 20-session course of TMS and all scheduled assessments were included in the final analysis. The mean (SD) age was 44.4 (17.5) years, and 70.8% of participants were female (Table 1). Thirty-one patients received HF-rTMS and 58 received iTBS.

**Table 1.** Demographic Data and Clinical Characteristics of Patients with Treatment-Resistant Depression Table 1. long description.

*n* (%) or mean ± SD	TRD (*n* = 89)			*P* for time
	Baseline	Day10	Day20	
Age, year	44.4 (17.5)			
Female	63 (70.8)			
Education, year	14.5 (2.7)			
Duration of illness	9.9 (11.5)			
rTMS: iTBS	31: 58			
Baseline				
HAMD total	23.08 (4.22)	11.42 (5.82)	8.75 (5.55)	< 0.001
HAMD item 3 (SI)	1.54 (1.02)	0.66 (0.76)	0.42 (0.61)	< 0.001
CGI-severity	5.35 (0.67)	3.20 (0.92)	2.41 (1.03)	< 0.001
Responders at day20, *N* (%)	58 (65.2)			
Remitters at day20, *N* (%)	46 (51.7)			
Antidepressants	81 (91.0)			
Antipsychotics	47 (52.8)			
Mood stabilizers	9 (10.1)			
Hypnotics or anxiolytics	72 (80.9)			

Abbreviation: CGI, clinical global impression; iTBS, intermittent theta-burst stimulation; rTMS, repetitive transcranial magnetic stimulation; SD, standard deviation; HAMD, 17-item Hamilton Depression Rating Scale; SD, standard deviation; SI, suicidal ideation.

Depressive symptoms improved significantly across the treatment course (Table 1). Mean HAMD-17 scores decreased from 23.08 (4.22) at baseline to 11.42 (5.82) at day 10 and 8.75 (5.55) on day 20 (*p* < 0.001 for time). Suicidal ideation and CGI-severity scores also showed significant reductions over time (both *p* < 0.001). Among treatment completers on day 20, 65.2% of patients met response criteria and 51.7% achieved remission.

Several indices of cognitive performance showed significant time effects across repeated assessments (Table 2). Working memory improved over time, reflected by increased correct responses and reduced omission errors in the 2-back task. Improvements in inhibitory control were observed through reductions in Go/No-Go commission errors. In the divided-attention task, visual commission errors decreased significantly across treatment, and auditory omission errors also showed a significant time effect.

**Table 2.** Performance of Neurocognitive Examinations of Patients with Treatment-Resistant Depression Table 2. long description.

mean ± SD	TRD (*n* = 89)			P for time
	Baseline	Day10	Day20	
1-back				
correct	13.86 (2.42)	14.22 (1.64)	14.48 (1.62)	0.065
commission error	1.08 (6.86)	0.31 (2.12)	0.06 (0.39)	0.362
omission error	1.14 (2.42)	0.78 (1.64)	0.52 (1.62)	0.065
2-back				
correct	10.45 (3.37)	11.15 (3.70)	11.62 (3.39)	0.011
commission error	1.42 (4.18)	1.08 (2.18)	0.78 (1.66)	0.267
omission error	4.55 (3.37)	3.85 (3.70)	3.38 (3.39)	0.011
Divided-attention visual				
correct	15.22 (2.96)	15.66 (2.79)	15.71 (1.97)	0.247
commission error	1.49 (4.67)	1.09 (5.35)	0.14 (0.43)	0.049
omission error	1.78 (2.96)	1.32 (2.77)	1.29 (1.97)	0.238
Divided-attention auditory				
correct	15.22 (1.71)	14.78 (2.32)	15.12 (2.09)	0.093
commission error	2.12 (9.57)	2.51 (9.71)	2.28 (11.08)	0.256
omission error	0.78 (1.71)	1.22 (2.32)	0.75 (1.52)	0.037
Go/No-Go				
correct	19.09 (2.34)	19.26 (2.09)	19.57 (1.22)	0.074
commission error	1.48 (2.67)	0.54 (1.31)	0.57 (1.21)	0.015
omission error	0.91 (2.34)	0.74 (2.09)	0.43 (1.22)	0.074

We first evaluated the overall time effect on depressive symptoms using a GEE model. HAMD-17 scores decreased significantly over time in model 1 (Table 3). Both HF-rTMS and iTBS protocols targeted left DLPFC and shared comparable antidepressant efficacy (Table 3). To assess the time-adjusted association between cognitive performance and depressive symptom severity, TAP indices were added as a time-varying covariate in model 2. After adjustment for multiple comparisons using the Benjamini–Hochberg FDR procedure, DAV_error was the only cognitive index that remained significantly associated with depressive symptom severity. Higher DAV_error was associated with higher HAMD-17 scores, while the time effect remained significant (day 10 vs baseline: *B* = −11.59, *p* < 0.001; day 20 vs baseline: *B* = −14.14, *p* < 0.001). An exploratory analysis examining the interaction between treatment modality and time was conducted. The time × treatment (HF-rTMS vs iTBS) interaction was not statistically significant (*p* = 0.058), suggesting that the trajectory of depressive symptom improvement did not differ significantly between patients receiving HF-rTMS and those receiving iTBS.

**Table 3.** Longitudinal Associations between Depressive Symptom Severity (HAMD Total Score) and Visual Divided Attention during Transcranial Magnetic Stimulation Treatment in the Generalized Estimating Equations Model Table 3. long description.

	Model 1			Model 2		
	*B*	Std. Error	*p*	*B*	Std. Error	*p*
Time: day 10 (vs baseline)	−11.66	0.77	<0.001	−11.59	0.74	<0.001
Time: day 20 (vs baseline)	−14.44	0.79	<0.001	−14.22	0.80	<0.001
Male	−2.56	0.82	0.002	−2.50	0.82	0.002
Age	0.000	0.03	0.995	−0.002	0.03	0.938
rTMS_iTBS	−1.60	0.86	0.061	−1.88	0.83	0.023
DAV_error				0.17	0.05	<0.001

To further clarify whether the observed association reflected between-person differences or within-person changes, a person-mean centering approach was applied. When both components were entered simultaneously into the GEE model, the within-person component of divided-attention visual errors remained significantly associated with depressive symptom severity (*B* = 0.209, *SE* = 0.036, *p* < 0.001), whereas the between-person component was not significant (*B* = 0.147, *SE* = 0.247, *p* = 0.551).

**Figure 2.**
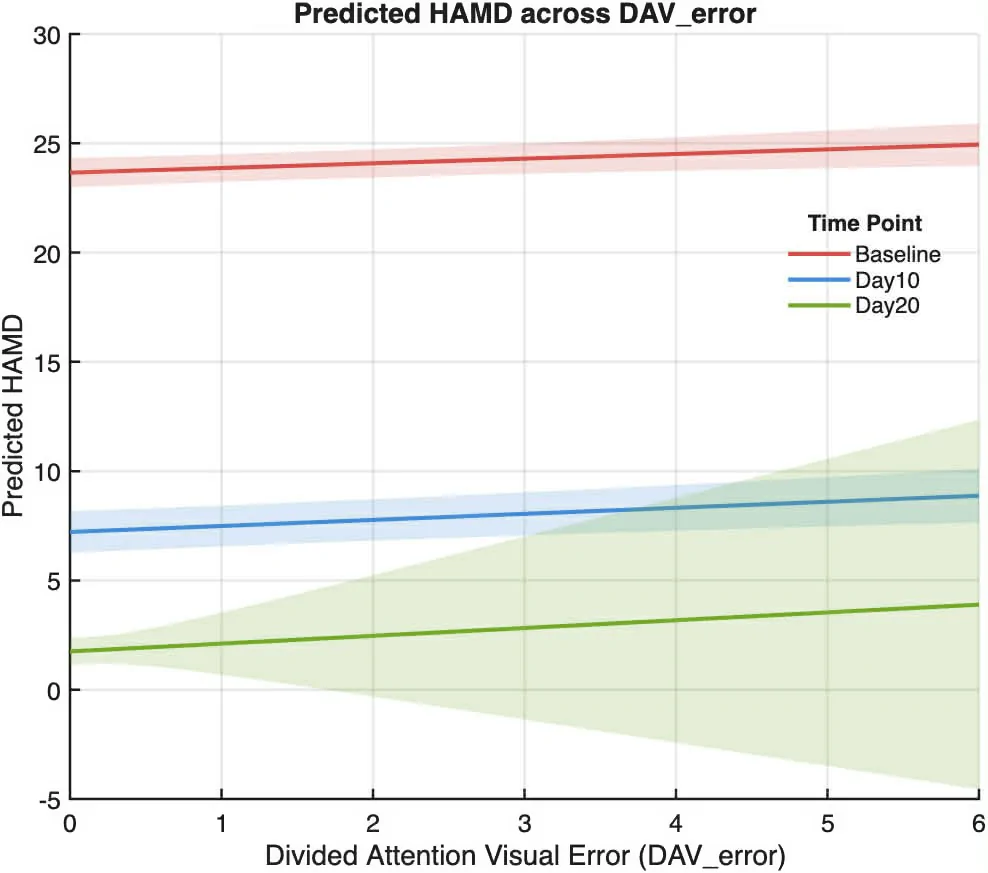
Time-adjusted association between visual divided-attention commission errors and depressive symptom severity during a 20-session TMS course. Predicted Hamilton Depression Rating Scale-17 item (HAMD-17) total scores were derived from generalized estimating equation models including time (baseline, day 10, day 20) and covariates (age, sex, and treatment modality [HF-rTMS vs iTBS]). Visual divided-attention commission errors (DAV_error) from the test of attentional performance were modeled as a time-varying predictor. Shaded areas indicate 95% confidence intervals. Confidence intervals were wider for the day 20 curve at higher DAV_error values, reflecting the smaller number of observations in that range. DAV_error indicates visual divided-attention commission errors; HF-rTMS, high-frequency repetitive transcranial magnetic stimulation; iTBS, intermittent theta-burst stimulation; TMS, transcranial magnetic stimulation. Figure 2. long description.

Model-based predicted HAMD-17 scores were generated across levels of DAV_error at baseline, day 10, and day 20 to visualize the time-adjusted association between attentional performance and depressive symptom severity (Figure 2). Across all time points, higher levels of DAV_error were consistently associated with higher predicted HAMD-17 scores.

Logistic regression analyses showed that none of the baseline TAP indices were significantly associated with response or remission at day 20. In an exploratory analysis examining the correlations between early cognitive change and subsequent symptom improvement, patients who showed greater early reduction in DAV_error demonstrated larger overall reductions in HAMD-17 scores across the treatment course (*B* = 0.172, *SE* = 0.070, *p* = 0.016).

## Discussion

In this retrospective, naturalistic study of patients with TRD who completed a 20-session course of left DLPFC TMS (HF-rTMS or iTBS), depressive symptoms improved over time. Several attentional control indices measured by the TAP also showed favorable time effects, including improved 2-back performance, fewer Go/No-Go commission errors, and fewer divided-attention errors. When cognitive indices were modeled as time-varying covariates within a longitudinal framework and corrected for multiple comparisons, only DAV_error remained independently associated with depressive symptom severity. Person-mean centering further indicated that this association was driven by within-person fluctuations rather than stable between-person differences. Baseline cognitive performance was not significantly associated with response or remission at day 20, whereas early improvement in DAV_error showed a modest association with greater overall symptom improvement.

Our findings align with the broader literature suggesting that TMS does not produce generalized cognitive enhancement in depression, with effects appearing small and task-specific rather than domain-wide. Meta-analytic work focusing on sham-controlled trials has most consistently identified modest benefits on tests emphasizing processing speed and set-shifting, while showing null effects across many other neuropsychological outcomes.^10^ ^–^^12^ The evidence base for attentional control paradigms closely overlapping with our TAP outcomes: working-memory updating, divided attention, and response inhibition, is sparse in the TMS literature in depression. When mapped onto specific constructs captured by our TAP battery, the prior randomized controlled trial (RCT) literature has been dominated by traditional neuropsychological proxies rather than repeated computerized tasks: working memory has most commonly been indexed by digit span measures; response inhibition by Stroop color–word interference; and attention/visual scanning and set-shifting by trail making test.^12^ In one sham-controlled trial of prefrontal deep TMS, cognitive outcomes included Delis–Kaplan executive function system (D-KEFS) color–word interference, a response-inhibition-related measure, and showed no advantage of active stimulation over sham, suggesting no robust cognitive enhancement.^22^ In that context, the time-related improvements we observed across multiple TAP indices should be interpreted cautiously because our design lacked a sham comparator and included repeated testing; practice effects and regression to the mean could contribute to gains. By contrast, our observation of working memory updating change on the 2-back task differs from the predominant sham-controlled depression literature using digit span, which has generally not shown reliable advantages of active TMS over sham for span-based working memory indices.^11^ ^,^^12^ One explanation is measurement sensitivity: n-back tasks involve continuous updating, monitoring, and interference control, reflecting dynamic executive demands that differ from simple storage-oriented span tasks.^23^ Electrophysiological and behavioral studies have shown that n-back engages frontal theta and P300 processes more strongly than simple span measures,^23^ and that n-back and span tasks correlate weakly,^24^ consistent with distinct cognitive requirements across tasks. Similarly, the improvement we observed in response inhibition should be interpreted alongside the fact that sham-controlled depression trials have more often used Stroop interference as an inhibitory-control proxy and have not consistently demonstrated TMS-specific benefits.^12^

A central finding was the parallel change between DAV_error and depressive symptom severity across repeated assessments: higher DAV_error tracked higher depressive symptom severity during the treatment course, and this association was explained by within-person fluctuations rather than stable between-person differences. Prior cross-modal attention studies have shown that divided attention between simultaneous auditory and visual events engages the DLPFC, supporting the view that this task captures a prefrontal cognitive control process.^18^ ^,^^25^ ^–^^27^ Experimental studies using rTMS in healthy participants have suggested that transient modulation of prefrontal regions can alter divided-attention performance.^18^ ^,^^27^ Our findings are broadly consistent with literature indicating that divided attention engages DLPFC-related cognitive control processes; however, in the present study, the results should be interpreted as showing parallel change during the treatment course rather than a direct effect of TMS on this cognitive domain. At the same time, this association should be interpreted cautiously. The effect size was modest relative to the much larger overall time-related reduction in HAMD-17 scores, indicating that DAV_error is not yet a definite state marker based on the present data. Rather, the current findings support a statistically detectable within-person coupling between depressive symptoms and divided-attention performance during TMS. Although greater early reduction in DAV_error was associated with greater overall HAMD improvement, this effect was also modest and exploratory.^28^ ^,^^29^ Mechanistically, repeated left DLPFC stimulation may influence divided attention by modulating frontoparietal cognitive control systems, reducing susceptibility to distraction and maladaptive internal focus, and possibly engaging downstream frontostriatal and monoaminergic pathways.^30^ ^–^^34^ However, these mechanistic interpretations remain inferential, as no neurophysiological or neuroimaging measures were acquired in the present study.

These results provide several contributions. By leveraging 3 repeated assessments during a standardized 20-session course, this study extends prior work that relied largely on pre–post comparisons and had limited ability to evaluate coupling between cognitive and clinical change.^10^ The identification of a time-adjusted, within-person association between divided-attention performance and symptom severity suggests that brief, repeatable attention-control measures may capture aspects of symptom–cognition dynamics during treatment. The exploratory association between early improvement in DAV_error and later symptom reduction further supports the possibility that early cognitive change could provide a signal of subsequent clinical improvement, although this requires prospective validation before clinical use.

A major methodological limitation of this study is that the analyses were restricted to a treatment-completer cohort. This design may introduce survivorship bias and may overestimate treatment effects compared with the full population initiating TMS. This attrition pattern differs from conventional loss to follow-up. In real-world TMS practice, treatment duration is strongly influenced by non-clinical factors, including out-of-pocket cost, daily time commitment, transportation burden, and shared clinician–patient decision-making. Standard TMS protocols require daily treatments over several weeks, which represents a substantial time commitment and limits treatment accessibility. Consistent with this, naturalistic cohort studies report substantial dropouts before completion of full treatment courses, highlighting adherence as a major implementation challenge in real-world TMS programs. Although baseline demographic and clinical characteristics, as well as day 10 response and remission rates, did not differ significantly between patients who completed 20 sessions and those who discontinued after 10 sessions, supplementary analyses showed significant group differences in several day 10 auditory divided-attention indices, suggesting possible differences in early cognitive trajectories between completers and non-completers. Thus, while the completer cohort did not appear to be selectively enriched for early clinical responders, some degree of cognitive selection bias cannot be excluded. Although the primary longitudinal finding involved DAV_error rather than auditory indices, the observed symptom–cognition coupling may preferentially reflect patients who were able or willing to continue treatment and therefore may not fully generalize to all patients initiating TMS. Nevertheless, unmeasured factors, such as financial constraints, logistical barriers, patient expectations, and perceived benefit, may still have influenced treatment continuation decisions. Future studies should model treatment adherence and dropout using intention-to-treat designs or joint longitudinal–survival approaches to better characterize real-world treatment trajectories.

This study has several important limitations. First, the retrospective, open-label design without a sham control precludes causal inference and increases susceptibility to selection bias, expectancy effects, and regression to the mean. The wide age range of the participants may have introduced heterogeneity in cognitive performance. Although age was adjusted for analytically, the small number of participants aged 65 years or older (*n* = 12) precluded adequately powered subgroup or age-by-time interaction analyses. Second, repeated cognitive testing limits the interpretability of observed cognitive time effects; improvements may partially reflect familiarity with tasks despite practice trials. Third, treatment modality heterogeneity (HF-rTMS vs iTBS) and nonrandom treatment selection may introduce confounding by indication. We adjusted treatment group in the analyses to address unmeasured determinants of treatment choice, and we found no significant difference between the 2 groups in depression severity trajectories.^35^ Fourth, cognitive assessment focused on attentional control tasks; episodic memory and broader executive measures were not systematically available, limiting comparison with prior task-level findings (eg, trail making test) and restricting inference to the measured domains. Fifth, the exploratory analysis relating early change in DAV_error to overall symptom improvement should be interpreted cautiously because baseline cognitive performance was not separately modeled, regression to the mean may have contributed, and the overlap between early and overall change scores may have introduced mathematical coupling.

In conclusion, among patients with TRD who completed a 20-session course of left DLPFC TMS in routine clinical care, depressive symptoms decreased over time and several attentional control indices showed time-related changes across repeated assessments. After accounting for time and multiple comparisons, within-person fluctuations in DAV_error were the cognitive measure most consistently associated with depressive symptom severity, and exploratory analyses suggested that early change in this measure may be associated with greater overall symptom reduction. Given the naturalistic, treatment-completer design and the absence of a sham comparator, these findings should be considered hypothesis-generating rather than evidence of TMS-related cognitive effects. Prospective, sham-controlled studies incorporating alternate test forms, intention-to-treat frameworks, and longitudinal outcomes are needed to determine whether divided-attention metrics represent a state-sensitive marker of cognitive control change during neuromodulation and whether such changes have prognostic or functional significance.

## Supporting information

10.1017/S1092852926100935.sm001 Huang et al. supplementary material Huang et al. supplementary material

## Data Availability

The data that support the findings of this study are available on request from the corresponding author. The data are not publicly available due to privacy or ethical restrictions.

## Supplementary material

The supplementary material for this article can be found at http://doi.org/10.1017/S1092852926100935.

## Figure 2. Long description

The x-axis is labeled Divided Attention Visual Error (D A V underscore error) ranging from 0 to 6. The y-axis is labeled Predicted H A M D, ranging from negative 5 to 30. Three lines represent time points: baseline in red, day 10 in blue, and day 20 in green. All lines show a positive linear trend, with higher D A V underscore error associated with higher predicted H A M D scores. The red baseline line starts near 24 and rises slightly to about 26 as D A V underscore error increases. The blue day 10 line starts near 7 and rises to about 10. The green day 20 line starts near 2 and rises to about 7. Shaded regions around each line indicate 95 percent confidence intervals, with the green day 20 interval widening substantially at higher D A V underscore error values. The legend at upper right identifies each line by color and time point.

Navigate back to Figure 2..

## Table 1. Long description

From the top row downward, the table lists demographic and clinical variables for 89 patients with treatment-resistant depression. The first column names each variable: age (mean 44.4 years, S D 17.5), female (63, 70.8 percent), education (mean 14.5 years, S D 2.7), duration of illness (mean 9.9 years, S D 11.5), r T M S to i T B S ratio (31 to 58), and medication categories. Baseline values are provided for each variable. For clinical outcomes, H A M D total scores decrease from 23.08 (S D 4.22) at baseline to 11.42 (S D 5.82) at day 10 and 8.75 (S D 5.55) at day 20, with P for time less than 0.001. H A M D item 3 (S I) scores decline from 1.54 (S D 1.02) at baseline to 0.66 (S D 0.76) at day 10 and 0.42 (S D 0.61) at day 20, P less than 0.001. C G I-severity scores drop from 5.35 (S D 0.67) at baseline to 3.20 (S D 0.92) at day 10 and 2.41 (S D 1.03) at day 20, P less than 0.001. Responders at day 20 number 58 (65.2 percent), remitters at day 20 number 46 (51.7 percent). Medication use at baseline includes antidepressants (81, 91.0 percent), antipsychotics (47, 52.8 percent), mood stabilizers (9, 10.1 percent), and hypnotics or anxiolytics (72, 80.9 percent). Abbreviations are defined below the table.

Navigate back to Table 1..

## Table 2. Long description

Starting from the top, the table lists five neurocognitive tests: 1-back, 2-back, divided-attention visual, divided-attention auditory, and Go/No-Go. Each test is subdivided into correct, commission error, and omission error rows. For the 1-back test, correct scores increase from 13.86 plus or minus 2.42 at Baseline to 14.48 plus or minus 1.62 at Day 20, with P for time 0.065. Commission errors decrease from 1.08 plus or minus 6.86 to 0.06 plus or minus 0.39, P for time 0.362. Omission errors decrease from 1.14 plus or minus 2.42 to 0.52 plus or minus 1.62, P for time 0.065. For the 2-back test, correct scores rise from 10.45 plus or minus 3.37 to 11.62 plus or minus 3.39, P for time 0.011. Commission errors drop from 1.42 plus or minus 4.18 to 0.78 plus or minus 1.66, P for time 0.267. Omission errors decrease from 4.55 plus or minus 3.37 to 3.38 plus or minus 3.39, P for time 0.011. Divided-attention visual correct scores increase slightly from 15.22 plus or minus 2.96 to 15.71 plus or minus 1.97, P for time 0.247. Commission errors decrease from 1.49 plus or minus 4.67 to 0.14 plus or minus 0.43, P for time 0.049. Omission errors decrease from 1.78 plus or minus 2.96 to 1.29 plus or minus 1.97, P for time 0.238. Divided-attention auditory correct scores fluctuate from 15.22 plus or minus 1.71 at Baseline to 15.12 plus or minus 2.09 at Day 20, P for time 0.093. Commission errors remain similar, from 2.12 plus or minus 9.57 to 2.28 plus or minus 11.08, P for time 0.256. Omission errors decrease from 0.78 plus or minus 1.71 to 0.75 plus or minus 1.52, P for time 0.037. Go/No-Go correct scores increase from 19.09 plus or minus 2.34 to 19.57 plus or minus 1.22, P for time 0.074. Commission errors decrease from 1.48 plus or minus 2.67 to 0.57 plus or minus 1.21, P for time 0.015. Omission errors decrease from 0.91 plus or minus 2.34 to 0.43 plus or minus 1.22, P for time 0.074. Statistically significant improvements are observed in correct scores and error reductions for 2-back, divided-attention visual commission errors, divided-attention auditory omission errors, and Go/No-Go commission errors.

Navigate back to Table 2..

## Table 3. Long description

From the top row downward, the table lists variables: Time day 10 versus baseline, Time day 20 versus baseline, Male, Age, r T M S underscore i T B S, and D A V underscore error. For each variable, values are provided for Model 1 and Model 2 in three columns: B, Standard Error, and p. For Time day 10, Model 1 B is minus 11.66, Standard Error 0.77, p less than 0.001; Model 2 B is minus 11.59, Standard Error 0.74, p less than 0.001. For Time day 20, Model 1 B is minus 14.44, Standard Error 0.79, p less than 0.001; Model 2 B is minus 14.22, Standard Error 0.80, p less than 0.001. For Male, Model 1 B is minus 2.56, Standard Error 0.82, p 0.002; Model 2 B is minus 2.50, Standard Error 0.82, p 0.002. For Age, Model 1 B is 0.000, Standard Error 0.03, p 0.995; Model 2 B is minus 0.002, Standard Error 0.03, p 0.938. For r T M S underscore i T B S, Model 1 B is minus 1.60, Standard Error 0.86, p 0.061; Model 2 B is minus 1.88, Standard Error 0.83, p 0.023. For D A V underscore error, only Model 2 values are shown: B 0.17, Standard Error 0.05, p less than 0.001. Negative B values for time and male indicate reductions in depressive symptom severity, with significant p values. D A V underscore error is positively associated in Model 2.

Navigate back to Table 3..
