# Supplementary material for: Longitudinal coupling between depressive symptoms and divided attention during transcranial magnetic stimulation in treatment-resistant depression
Source: CNS Spectr. 2026 May 13;31(1):e34. doi: 10.1017/S1092852926100935 (PMC13732622; doi:10.1017/S1092852926100935)
Supplement: Supplementary file 1 [file S1092852926100935sup001.docx]

Supplementary method

Neuropsychological assessment

Working memory was evaluated using the TAP 1-back and 2-back tasks. Participants were instructed to respond as quickly and accurately as possible when the currently presented number matched the one presented one trial earlier (1-back) or two trials earlier (2-back). Sustained attention and inhibitory control were assessed using the Go/No-Go task. Participants were instructed to respond as quickly as possible when the “×” symbol appeared and to withhold responses when the “+” symbol appeared. Commission errors were interpreted as impaired inhibitory control and impulsivity, whereas omission errors reflected deficits in sustained attention. Divided attention was assessed using simultaneous visual and auditory tasks. In the visual task, a 4×4 matrix containing 16 dots was displayed, and participants responded when four dots forming a square were marked with an “x.” In the auditory task, participants detected irregularities in sequences of high- and low-pitched tones.

Table 1. Comparisons of demographic data and clinical characteristics between patients who completed 10 sessions and those who continued to 20 sessions

|  | 20 session completers (n =89) | | 10 session completers (n =196)* | | P for baseline | P for day10 |
| --- | --- | --- | --- | --- | --- | --- |
| n (%) or mean ± SD | Baseline | Day10 | Baseline | Day10 |  |  |
| Age, year | 44.4 (17.5) |  | 46.2 (18.0) |  | 0.707 |  |
| Female | 63 (70.8) |  | 125 (63.8) |  | 0.282 |  |
| Education, year | 14.5 (2.7) |  | 14.3 (2.9) |  | 0.672 |  |
| Duration of illness | 9.9 (11.5) |  | 10.2 (9.9) |  | 0.746 |  |
| rTMS : iTBS | 31 : 58 |  | 75 : 121 |  | 0.599 |  |
| Baseline |  |  |  |  |  |  |
| HAMD total | 23.08 (4.22) | 11.42 (5.82) | 23.25 (4.15) | 12.76 (6.92) | 0.962 | 0.150 |
| HAMD item 3 (SI) | 1.54 (1.02) | 0.66 (0.76) | 1.46 (0.97) | 0.68 (0.84) | 0.566 | 0.773 |
| CGI-severity | 5.35 (0.67) | 3.20 (0.92) | 5.43 (0.57) | 3.03 (1.16) | 0.455 | 0.331 |
| Responders on day10, N (%) |  | 43 (48.3) |  | 89 (45.4) |  | 0.701 |
| Remitters on day10, N (%) |  | 29 (32.6) |  | 76 (38.8) |  | 0.355 |
| 1-back |  |  |  |  |  |  |
| correct | 13.86 (2.42) | 14.22 (1.64) | 13.83 (2.51) | 13.77 (2.56) | 0.947 | 0.211 |
| commission error | 1.08 (6.86) | 0.31 (2.12) | 1.08 (4.02) | 0.52 (2.18) | 0.989 | 0.530 |
| omission error | 1.14 (2.42) | 0.78 (1.64) | 1.17 (2.51) | 1.23 (2.56) | 0.947 | 0.211 |
| 2-back |  |  |  |  |  |  |
| correct | 10.45 (3.37) | 11.15 (3.70) | 10.13 (3.75) | 10.38 (3.80) | 0.615 | 0.210 |
| commission error | 1.42 (4.18) | 1.08 (2.18) | 1.99 (6.52) | 0.99 (2.63) | 0.537 | 0.789 |
| omission error | 4.55 (3.37) | 3.85 (3.70) | 4.87 (3.75) | 4.61 (3.79) | 0.615 | 0.213 |
| Divided attention visual |  |  |  |  |  |  |
| correct | 15.22 (2.96) | 15.66 (2.79) | 15.49 (2.33) | 15.53 (2.18) | 0.454 | 0.773 |
| commission error | 1.49 (4.67) | 1.09 (5.35) | 0.85 (2.04) | 0.47 (1.22) | 0.153 | 0.176 |
| omission error | 1.78 (2.96) | 1.32 (2.77) | 1.50 (2.33) | 1.47 (2.18) | 0.443 | 0.726 |
| Divided attention auditory |  |  |  |  |  |  |
| correct | 15.22 (1.71) | 14.78 (2.32) | 14.94 (2.25) | 15.32 (1.24) | 0.414 | 0.027 |
| commission error | 2.12 (9.57) | 2.51 (9.71) | 1.39 (4.73) | 0.79 (2.87) | 0.439 | 0.048 |
| omission error | 0.78 (1.71) | 1.22 (2.32) | 1.03 (2.23) | 0.68 (1.24) | 0.462 | 0.027 |
| Go/No-Go |  |  |  |  |  |  |
| correct | 19.09 (2.34) | 19.26 (2.09) | 18.83 (3.30) | 19.39 (1.71) | 0.594 | 0.605 |
| commission error | 1.48 (2.67) | 0.54 (1.31) | 1.65 (2.68) | 0.86 (1.24) | 0.713 | 0.098 |
| omission error | 0.91 (2.34) | 0.74 (2.09) | 1.17 (3.30) | 0.61 (1.71) | 0.594 | 0.605 |

CGI: clinical global impression; iTBS: intermittent theta-burst stimulation; rTMS: repetitive transcranial magnetic stimulation; SD: standard deviation; HAMD: 17-item Hamilton Depression Rating Scale; SD: standard deviation; SI: suicidal ideation

*A total of 210 patients with treatment-resistant depression completed 10 sessions of TMS and underwent interviews and clinical ratings. However, because the cognitive assessments were time-consuming, four patients did not complete the full cognitive test battery and were excluded from the analysis.
